# Combined Raman and polarization sensitive holographic imaging for a multimodal label-free assessment of human sperm function

**DOI:** 10.1038/s41598-019-41400-0

**Published:** 2019-03-18

**Authors:** Annalisa De Angelis, Maria Antonietta Ferrara, Gianfranco Coppola, Loredana Di Matteo, Laura Siani, Brian Dale, Giuseppe Coppola, Anna Chiara De Luca

**Affiliations:** 1 Institute of Protein Biochemistry, National Research Council of Italy, Via P Castellino 111, Naples, 80131 Italy; 2 Institute for Microelectronic and Microsystems, Unit of Naples, National Research Council of Italy, Via P Castellino 111, Naples, 80131 Italy; 3 Centro Fecondazione Assistita (CFA-Italia), Via Manzoni 15, Naples, 80123 Italy

## Abstract

Raman microspectroscopy (RM) and polarization sensitive digital holographic imaging (PSDHI) are valuable analytical tools in biological and medical research, allowing the detection of both biochemical and morphological variations of the sample without labels or long sample preparation. Here, using this multi-modal approach we analyze *in vitro* human sperm capacitation and the acrosome reaction induced by heparin. The multimodal microscopy provides morphofunctional information that can assess the sperms ability to respond to capacitation stimuli (sperm function). More precisely, the birefringence analysis in sperm cells can be used as an indicator of its structural normality. Indeed, digital holography applied for polarization imaging allows for revelation of the polarization state of the sample, showing a total birefringence of the sperm head in non-reacted spermatozoa, and a birefringence localized in the post-acrosomal region in reacted spermatozoa. Additionally, RM allows the detection and spectroscopic characterization of protein/lipid delocalization in the plasma and acrosomal membranes that can be used as valuable Raman biomarkers of sperm function. Interestingly, these spectral variations can be correlated with different time phases of the cell capacitation response. Although further experimentation is required, the proposed multimodal approach could represent a potential label-free diagnostic tool for use in reproductive medicine and the diagnosis of infertility.

## Introduction

Sperm cell capacitation is an essential step in the fertilization process; consequently it represents one of the most important aspects of semen assessment^1,2^. Indeed, sperm function tests aim to determine whether and how spermatozoa respond to capacitation stimuli, since only after capacitation, spermatozoa become competent to fertilize the oocyte. In natural fertilization, ejaculated spermatozoa capacitate in the female genital tract following a series of biochemical modifications that can be summarized as follows^3^. (i) The trigger event is the removal of the decapacitation factors (i.e. glycoproteins)^4^. These proteins are present in the epydidimal fluid and in the seminal plasma, stabilizing the sperm membrane and keeping spermatozoa in a non-capacitated state along the lower female tract. This event is accompanied by a time-dependent increase in protein tyrosine phosphorylation^5–7^. (ii) The formation of lipid rafts at the apical ridge of the sperm head containing sphingolipids, cholesterol and epididymal proteins^4^. Two types of proteins are mainly involved in membrane raft formation: caveolin and flotillin^8^. (iii) Later, cholesterol is oxidized and removed from the sperm surface by albumin. Cholesterol efflux facilitates the uptake of calcium ions that triggers the acrosome reaction^4^. At the cytosolic site, the apical ridge of the sperm head is now efficiently and stably docked to the outer acrosomal membrane. Before entering the oocyte, the acrosomal reaction occurs and the acrosome membrane, covering almost 50% of the nucleus of the sperm head, will fuse with the nucleus of the oocyte and the two pronuclei are formed indicating that fertilization is successful and this will then lead to the formation of the embryo^9^.

In assisted reproductive techniques (ARTs), sperm selection is a very important step to achieve pregnancy; in particular Mansour *et al*.^10^ demonstrated that better development of embryos is achieved by selecting reacted spermatozoa. However in ART, the natural sperm selection steps are bypassed, and capacitation is induced *in vitro*, before insemination, by using substances simulating the physiological conditions of the female tract, e.g. heparin^11,12^. Spermatozoa incubated with heparin are subjected to a cascade of events leading to specific modifications of the lipid/protein content and localization in the acrosomal region, mainly involving the plasma and outer acrosomal membranes, as described above.

Sperm function is normally evaluated using destructive and invasive protocols, such as the Chlorotetracycline (CTC) staining assay^13^ and the fluorescein-conjugated Pisum sativum agglutinin (PSA) assay^14^ that have the potential to assist in clinical decisions, however rendering the analyzed spermatozoon unsuitable for intracytoplasmic sperm injection (ICSI) especially for patients with an abnormally low sperm count. Recently, Moody and colleagues validated a Cap-Score Sperm Function Test^15^ based on the analysis of the glycolipid (monosialotetrahexosylganglioside GM1) localization patterns, reflecting the capacitated state in human sperm. Although these assays may classify an infertile man according to the type and degree of his spermatogenetic defect, these protocols provide limited information about how well a sperm will function in *in vitro* or *in vivo* settings. In this context, the birefringence of the spermatozoons head could be an interesting parameter to consider. It has been demonstrated that sperms heads with total birefringence have a normal structure of both the nucleus and the acrosome and might increase the probability for success in ICSI^16,17^. Owing to longitudinally oriented protein filaments, human sperm cells are naturally birefringent when observed under polarized light, while after the acrosome reaction, the local protein organization disaggregates leading to loss of the birefringence in the acrosomal region (the acrosome covers 40–70% of the sperm head)^18^; therefore, reacted spermatozoa show a partial head birefringence^19^, typically in the post-acrosomal region. Moreover, Magli *et al*.^20^ studied the birefringence in the sperm head and they classified the results in three groups, depending on the birefringent pattern: (i) spermatozoa with a totally birefringent head; (ii) spermatozoa with a partial birefringence localized in the post-acrosomal region; (iii) sperm with an irregular pattern of birefringence due to absence of birefringence in their heads, or the presence of vacuoles or small areas of birefringence confined either to the nuclear or acrosomal region^20^. Thus the possibility to distinguish between spermatozoa that are totally birefringent (acrosome intact, non-reacted spermatozoa) and those in which birefringence occurs only in the post-acrosomal region (reacted spermatozoa) is a parameter of clinical relevance. Unfortunately, sperm cells with partial birefringent heads has been revealed and correlated with other cell characteristics^20^. Thus, it is important to develop complementary protocols allowing non-invasive and non-destructive identification of sperm function at the cellular and molecular levels.

By combining polarization sensitive digital holographic imaging (PSDHI) with Raman Microscopy (RM), a more complete assessment of semen can be obtained^21–24^. RM and holographic imaging (HI) have already seen successfully applied for the label-free characterization of biological samples^25–27^, including the assessment of sperm cell physiology and morphology^28–30^. Indeed, HI, by analyzing the interference of the light passing through the object with the reference beam, was used to study cell and tissue imaging^31^, sperm cell morphology and volume^21–23^, including detection and measurements of sperm cell defects (such as the presence of vacuole) and motility^32,33^. Additionally, PSDHI based on the analysis of both amplitude and phase of the diffracted beam by means of two orthogonally polarized reference waves^34,35^, provides, together with the morphology and dynamics, intrinsic information about the polarization state of the sample through the phase change quantification. Therefore, polarized HI allows the study of birefringence of anisotropic samples^36^ such as the spermatozoon head. On the other hand, RM is a spectroscopy-based technique that measures inelastic scattering of light by vibrating molecules in cells^37,38^. The characteristic bands in Raman spectra are relatively narrow, easy to resolve, specific to molecular structure, conformation and environment. In this manner, the Raman ‘signature’ can be used to assess physiological status or to detect altered molecular features in sperm cells^39,40^. Indeed, RM has been used to detect protein denaturation, DNA fragmentation and lipid peroxidation in human sperm cells^30,39,41,42^.

In this paper, we propose to use multimodal RM and PSDHI for the label-free detection of the birefringence, morphology and Raman spectra of fixed sperm cells and the morphofunctional analysis of cell capacitation induced by heparin. Since during capacitation sperm cells undergo a series of structural and functional modifications essential for obtaining fertilizability, as previously described, in this work we show the ability of our optical approach to identify capacitated from uncapacitated sperm cells on the basis of these changes. Basically, the PSDHI is used for a rapid analysis of the distribution of birefringence, indicating total birefringence of the head in non-reacted spermatozoa, and a birefringence localized to the post-acrosomal region in reacted spermatozoa. More interestingly, RM results highly sensitive for revealing spectroscopic variation associated with the loss of surface proteins, protein phosphorylation, and in localizing the cholesterol efflux in the sperm plasma membrane. Indeed, these cell changes represent the capacitation triggering events and, thus, allow the identification of capacitated and non-capacitated spermatozoa with high reproducibility. Finally, we selected the reacted sperm cells on the basis of the preliminary rapid results obtained by PSDHI analysis. A complete analysis of the selected cells was performed in a very specific way, using RS, to follow the biochemical processes involved in the acrosome reaction, with the aim of determining if the detected changes in polarization can be correlated with the acrosome reaction.

## Results

### CTC fluorescence assay for sperm capacitation

**Figure 1:**
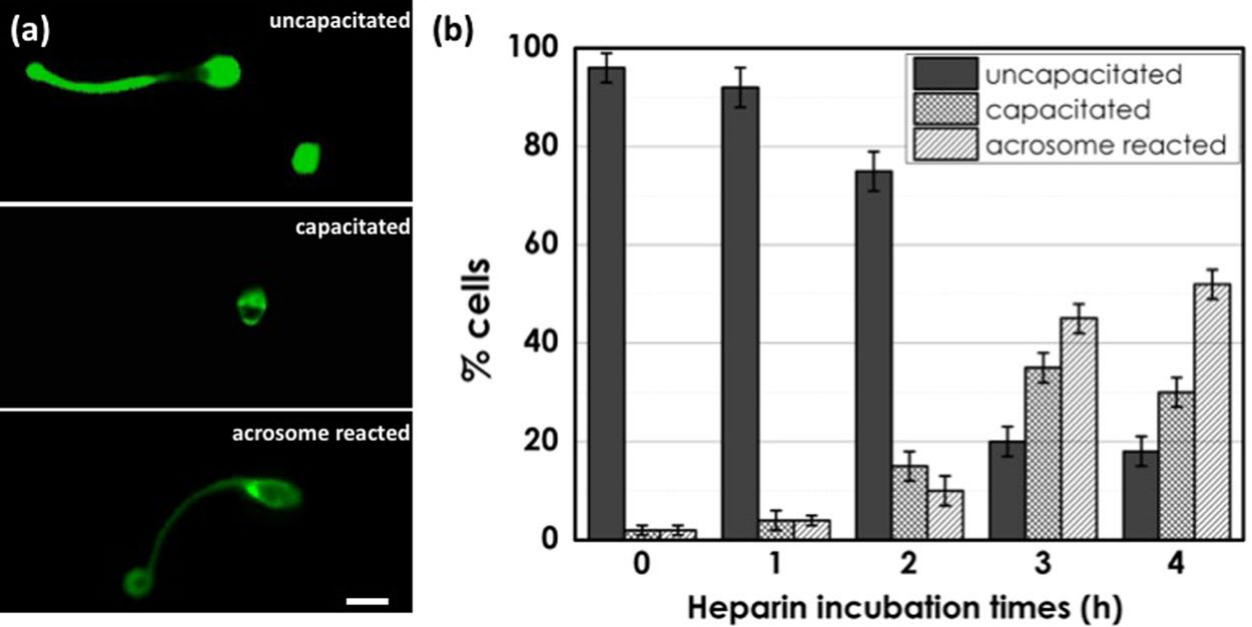
(**a**) Different stages of CTC stain of spermatozoa during the process of capacitation. Scale bar: 2 *μ*m. (**b**) Distribution of various CTC fluorescence patterns of sperm from 3 donors exposed to heparin for 0 (control sample), 1, 2, 3 and 4 hours.

Sperm cells from healthy donors exposed to heparin for different times (1, 2, 3 and 4 hours) were prepared, separated in two different aliquots, and their capacitation was analyzed. The CTC fluorescence assay, measuring the calcium levels in sperm cells, was performed on each heparin exposed sample. CTC-staining patterns were classified into three types according to the method described by Fraser *et al*.^43,44^: uncapacitated-spermatozoa, bright fluorescence detected over the whole region of the sperm head; capacitated-spermatozoa, fluorescence detected on the sperm head but not over the post-acrosomal region; and acrosome-reacted-spermatozoa, weak fluorescence observed over the sperm head with a bright band sometimes present in the equatorial region (Fig. 1a). The average percentages of sperm displaying the various CTC fluorescence patterns for all the samples are reported in Fig. 1b. The data show that for up to 2 hours of incubation with heparin, the uncapacitated spermatozoa were ≥75% of the population. After 3 h of treatment, CTC assay resulted in a higher value for the percentage acrosome-reacted/capacitated spermatozoa (about 80%). The percentage of acrosome-reacted/capacitated spermatozoa remained almost constant after 4 h (about 80%). The same incubation times have been evaluated using the multimodal approach.

### Polarization sensitive digital holographic imaging of the sperm capacitation

**Figure 2:**
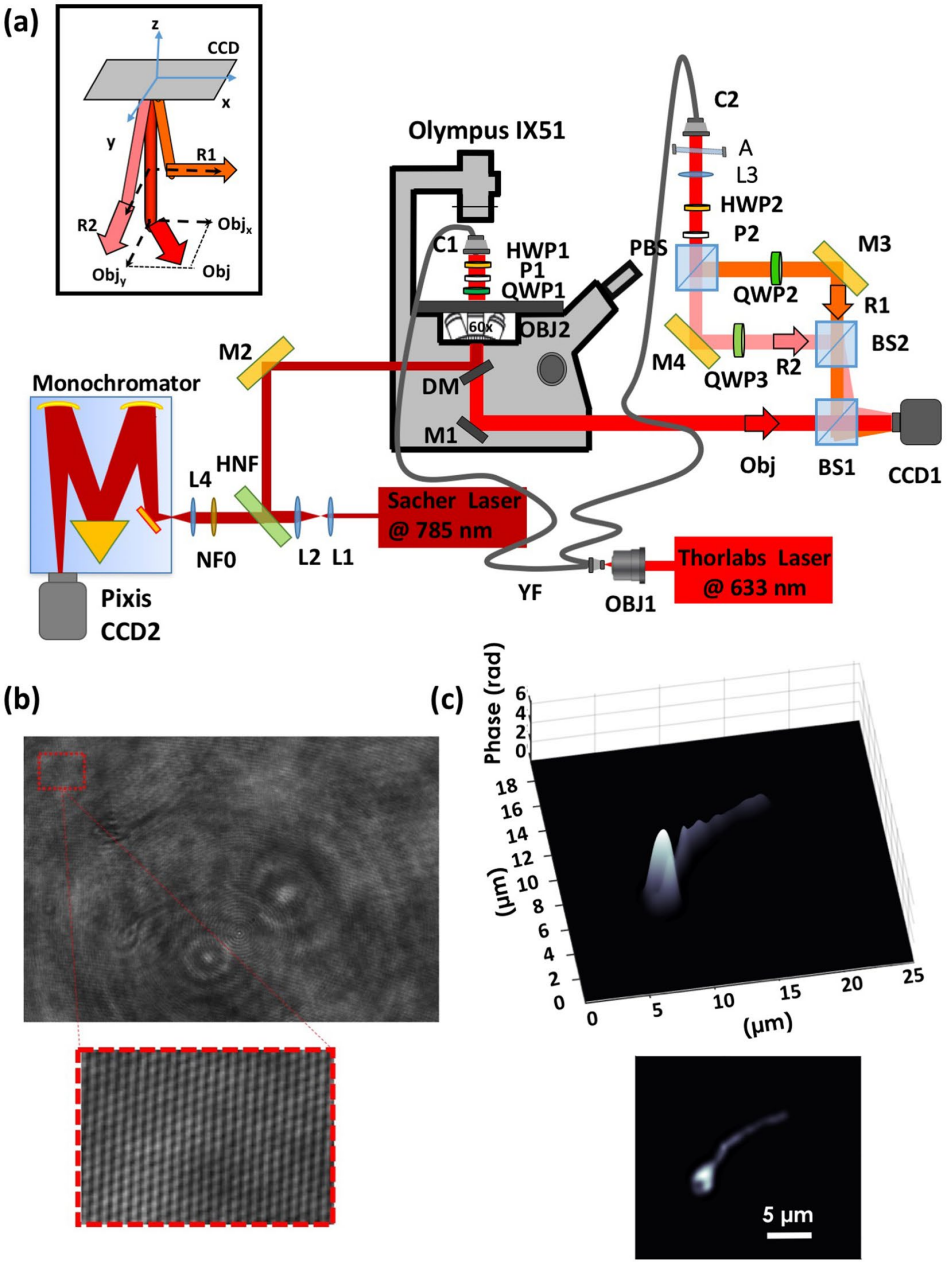
(**a**) Multimodal polarization sensitive digital holographic and Raman microscope experimental setup. In the inset, the incident directions and the polarization states of the object and reference waves are highlighted. Abbreviations: M, mirrors; L, lens; BS, beam splitter; PBS, polarized beam splitter; NF, notch filter; HNF, holographic notch filter; DM, dichroic mirror; QWP, quarter-wave plate; HWP, half-wave plate; C, collimator; YF, fiber Y-splitter. (**b**) Polarization hologram. The inset shows the intensity of the fringe pattern. (**c**) Calculated phase map for one of the two polarizations recorded and the 2D view.

The birefringence of the sperm cell samples was analyzed using the PSDHI setup, shown in Fig. 2a and described in the Materials and Methods section. A typical polarization hologram is reported in Fig. 2b where two different sets of fringe patterns are clearly visible (see the inset), due to the interference of the object field with the two orthogonal reference waves. The reconstructed three-dimensional phase map for one of the two polarizations is showed in Fig. 2c. Considering that the polarization state in sperm cells is expected to be different only between the control (intact acrosome) and reacted sperm cells^20^, thirty spermatozoa were investigated for control (0 h in heparin) and reacted sperm cells (4 h in heparin), respectively. The experiments have been repeated on three different donors.

**Figure 3:**
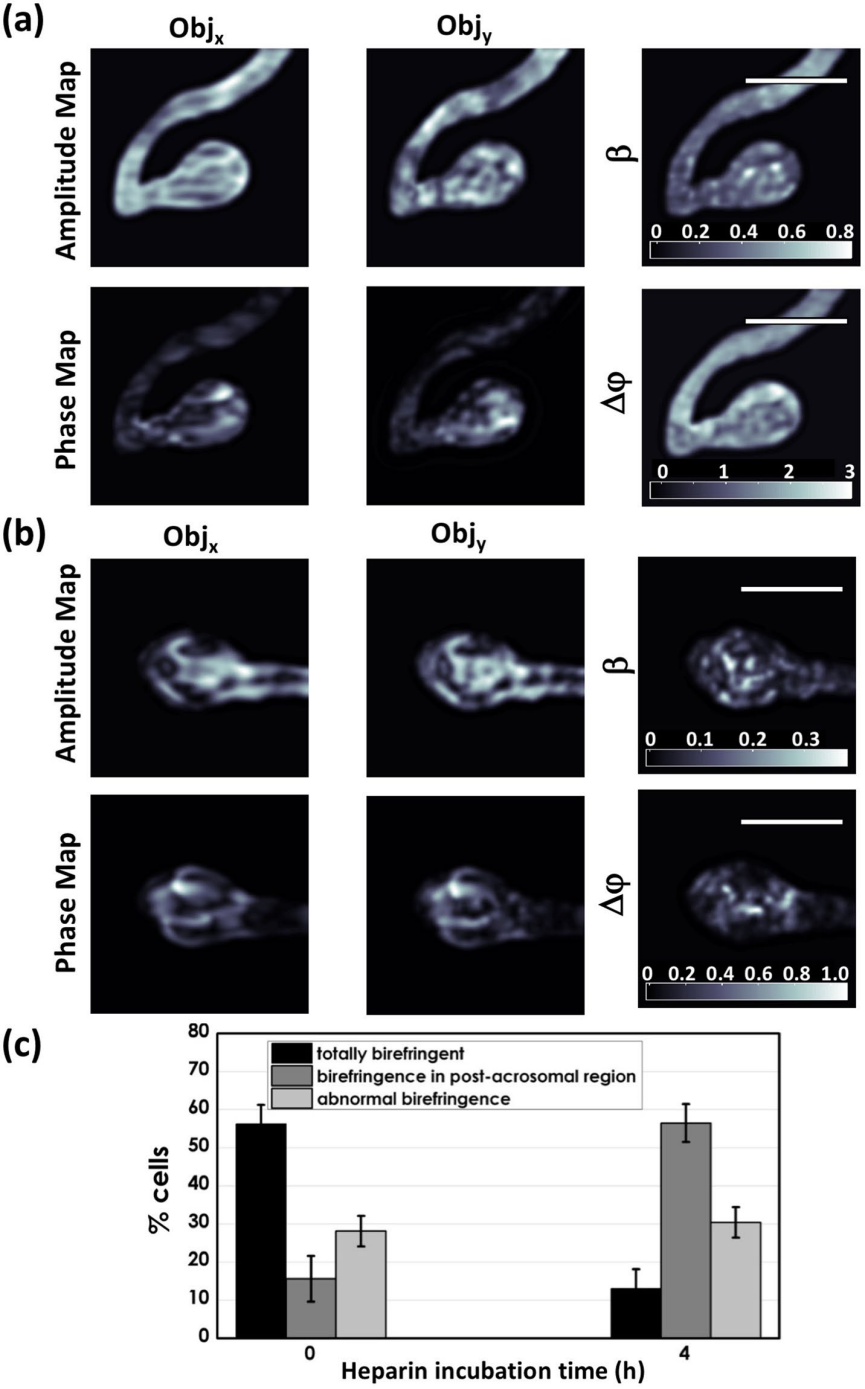
(**a**) Amplitude map of *Obj*_*x*_, and *Obj*_*y*_, amplitude parameter *β*, phase contrast for *Obj*_*x*_ and *Obj*_*y*_, phase difference Δ*ϕ* relative to a control sperm cell (0 h in heparin). Colorbars indicate the mapping of the phase variation. The Δ*ϕ* map shows a birefringence distribution (bright pattern) over all the sperm head. (**b**) The same holographic parameters measured for a reacted sample (4 h in heparin). The Δ*ϕ* map after 4 h in heparin shows a clear decrease of the birefringent area. In particular, the birefringence (bright pattern) is confined in the post-acrosomal region. (**c**) Distribution of birefringence patterns of sperm from 3 donors exposed to heparin for 0 h (control sample), and 4 h (reacted sample). Scale bar: 4 *μ*m.

An example of reconstructed amplitude maps and phase maps for the two components of the object field from one polarized hologram acquired on a control sperm cell is reported in Fig. 3a. Interestingly, the amplitude ratio and the phase difference parameters evaluated by using Eq. 2, reported in the Materials and Methods section and which are two characteristics of the Jones vector of the object wave^34^, clearly show a discontinuity in correspondence of the sperm head with respect to the background, due to a difference between the two orthogonal components, thus revealing the birefringence of the head of the sperm cell. The same analysis has been carried out for reacted sperm cells, and an example is showed in Fig. 3b. In this case, the birefringence pattern related to the discontinuity in both the amplitude ratio and the phase difference maps, is localized only in the post-acrosomal region, as expected for reacted samples^20^.

**Figure 4:**
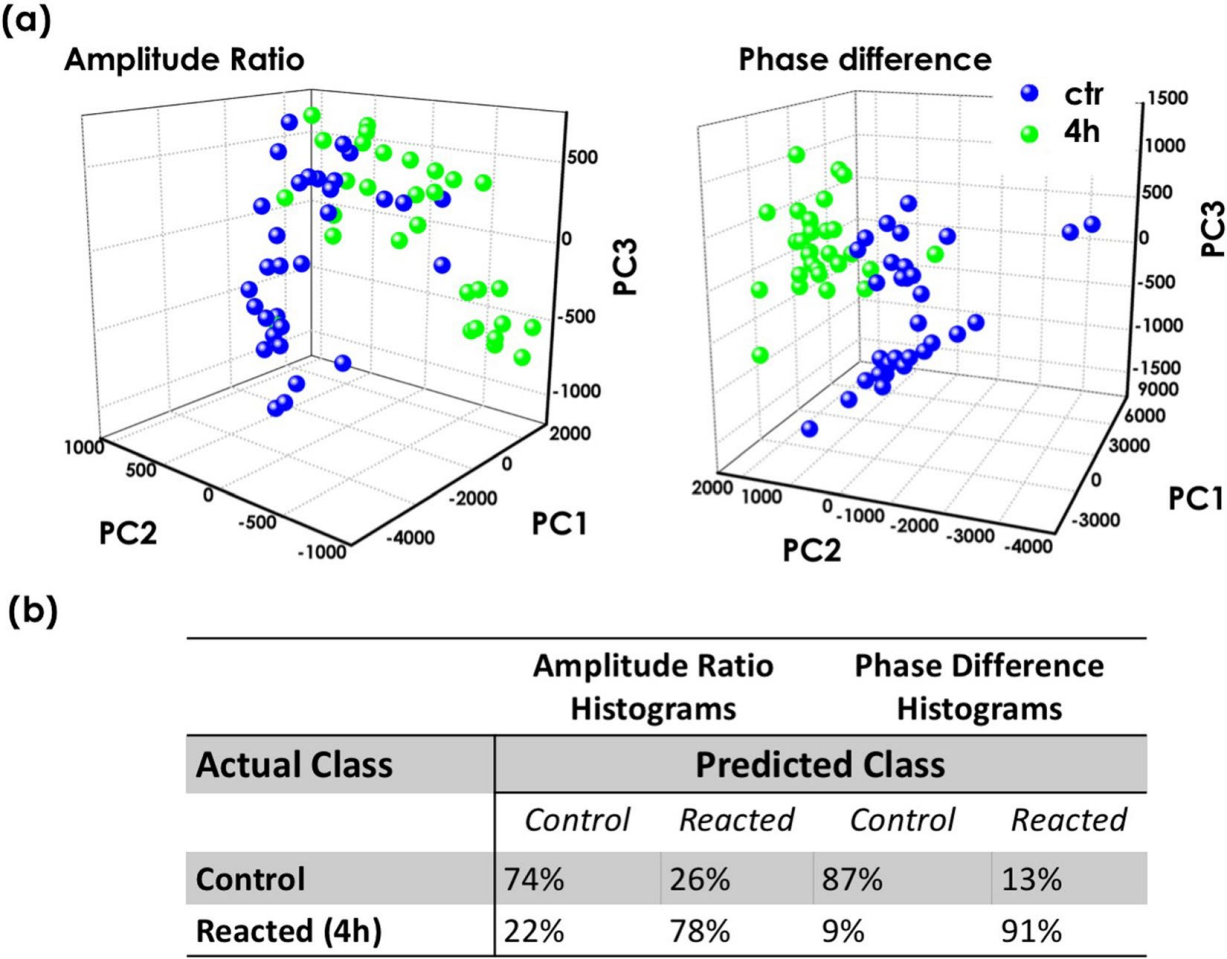
(**a**) 3D PCA scatter plots of the first three principal components for datasets obtained by analysing the amplitude ratio and the phase difference histograms. (**b**) Confusion matrix illustrating the efficiency of PSDHI to discriminate between control (0 h in heparin) and reacted (4 h in heparin) sperm cells.

A first result was obtained simply by examining the birefringence pattern by visual inspection, considering both the amplitude ratio and the phase difference maps for all analyzed sperm cells, and in relation to the three birefringence patterns (total, partial or abnormal), obtaining the distribution summarized in Fig. 3c. When reacted sperm cells are characterized, we observed a very high increase in the percentage of analyzed samples that show birefringence localized in post-acrosomal region accompanied by an evident decrease of sperm with the whole birefringent head respect to the control sample (intact acrosome). Additionally, we note a lower amount of samples with abnormal birefringence, i.e., with birefringence localized only in the acrosome or in the tail, and a very low number of sperm cells that are not birefringent. Those behaviours have been ascribed to the loss of motility and to the presence of abnormal morphology^20^. However, the low number of cells with irregular birefringence (≈28% in the control samples, and ≈30% in the reacted samples) ensures that  the spermatozoa  analyzed are for the most part motile and morphologically normal^20^. These results are in agreement with the percentage of non-motile spermatozoa obtained with the motility test (see Supplementary Information). A more precise differentiation between control and reacted samples was obtained by performing a statistical analysis directly on both the amplitude ratio and the phase difference maps. Due to the relatively low number of samples and in order to directly compare the results obtained with PSDHI and those obtained with RM, data were analysed by using Principal Component Analysis (PCA)^25^. The PCA analysis was performed on the whole data set using the first three principal components (PC1, PC2, PC3), which accounted for about 48%, 21% and 12% of the total variance for the phase difference Δ*ϕ* and about 81%, 8% and 7% of the total variance for the amplitude parameter *β*. The PCs loadings are reported in the supplementary information (see Fig. S1). Results can be seen in Fig. 4a, for the datasets obtained by analysing the amplitude ratio and the phase difference histograms, respectively. In detail, a mask was created on these maps in order to select as region of interest (ROI) only the sperm head, where the main variations associate with the acrosomal reaction occur^19,20^. Then, a histogram was generated to summarize the number of pixels at specific grey level values across the amplitude ratio and the phase difference masked images. These histograms allow to recognize the spread pixel values, indicating uniformity across the considered images. Representing these images in terms of their pixel intensity histogram provided two arrays of values and on each of them we performed PCA to discriminate between the control (0 h in heparin) and reacted (4 h in heparin) cell subsets. The leave-one-out cross validation approach has been used to build up the confusion matrix reported in Fig. 4b, showing excellent discrimination efficiencies. Indeed, we demonstrate that the sensitivity achieved increases from 82% for amplitude ratio to 93% for phase difference, while the specificity grows from 69% to 88%. Considering these results, appears clear that if we consider only the amplitude ratio, which can be obtained also with a standard polarized microscope, a good discrimination between control (0 h in heparin) and reacted (4 h in heparin) samples can be obtained, as already reported in literature^19,20^. However, the possibility to achieve phase information allowed by the proposed PSDHI microscope, and thus to evaluate the phase difference distribution in sperm cells, significantly increases the classification rate.

### Raman analysis of the sperm capacitation

**Figure 5:**
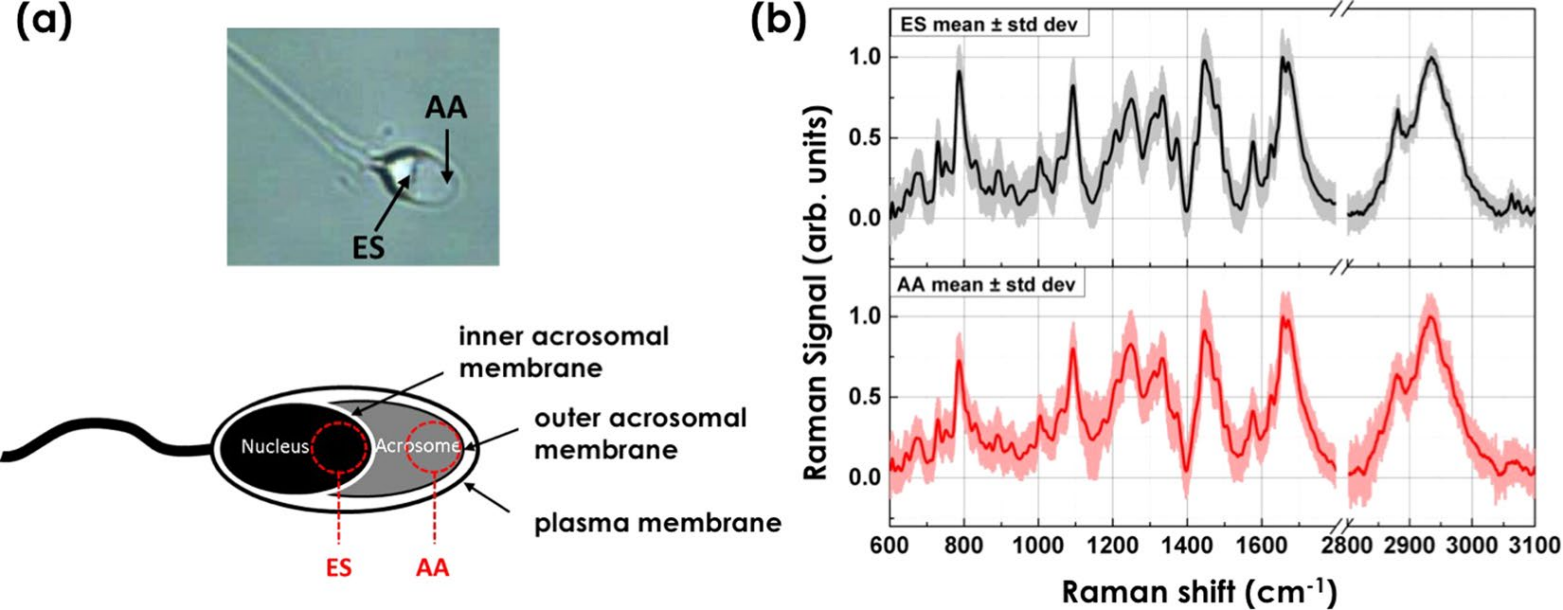
(**a**) Bright-field image and scheme of sperm cells from a healthy donor. Two regions of the spermatozoon head are mainly involved in the capacitation: the equatorial segment (ES) and the apical acrosomal vesicle (AA). (**b**) Mean Raman spectra from thirty cells from the selected head regions.

**Figure 6:**
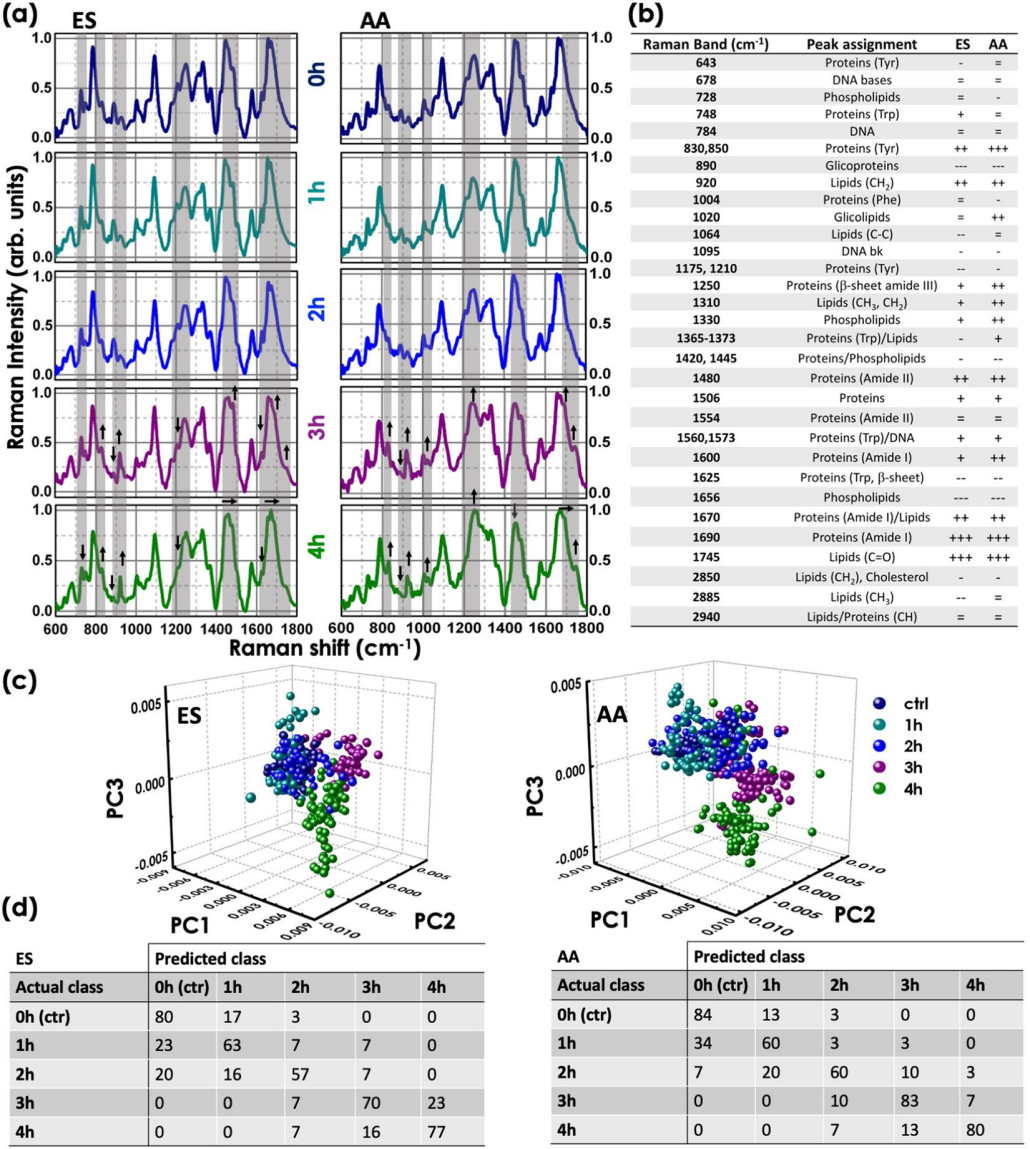
(**a**) Mean Raman spectra from the equatorial segment, ES, and the apical acrosomal vesicle, AA, of the sperm head after the treatment with heparin for 0 (control sample), 1, 2, 3 and 4 hours of incubation time. (**b**) Band assignments in Raman spectra from the equatorial segment (ES) and the apical acrosomal vesicle (AA), for non-capacitated and capacitated sperm cells. (**c**) 3D PCA scatter plots of the first three principal components for each the sperm cell region exposed to heparin for 0, 1, 2, 3 and 4 h. (**d**) Confusion matrix resulting from the cross validation procedure. The data reported in the table are expressed in %.

Since the equatorial segment (ES in Fig. 5a) and the apical acrosomal vesicle (AA in Fig. 5a) of the sperm head are mainly involved in the capacitation dynamics, we acquired and compared Raman spectra from these two regions. Thirty spermatozoa from 3 different healthy donors were investigated and the mean spectra and the standard deviation acquired from the two selected regions are reported in Fig. 5b. Both the spectra show a good reproducibility and signal-to-noise ratio and they are dominated by Raman bands associated with protein/lipid content. The extended peak assignment of the Raman bands are given in the table reported in Fig. 6. Indeed, the bands at 643, 830, 850, and 1210 cm^−1^ have been previously assigned to vibrational modes of tyrosine residues. The spectral range between 1200–1600 cm^−1^ is generally ascribed to proteins/lipids (CH and CH _2_ molecular vibrations), and, in more details, the peak at 1250 cm^−1^ is assigned to the *β*-sheet of amide III, the bands at 1310, 1330, 1625 and 1745 cm^−1^ are due to lipids and phospholipids, the bands at 1360–1450 cm^−1^ are linked to protein/lipid complex, the bands 1480 and 1554 cm^−1^ have been previously assigned to the amide II, and the peaks 1609, 1670 and 1690 cm^−1^ are due to the amide I. The high wavenumber spectral region (2850–3100 cm^−1^), arising from CH_2_ and CH_3_ stretching vibrations, further highlights the lipid contributions to the AA and ES Raman spectrum. Although Raman bands associated with proteins/lipids are similar in both the investigated regions, we can notice more intense peaks related to DNA structures, such as the bands in the region between 600–800 cm^−1^ corresponding to DNA bases, the band at 1095 cm^−1^ arising from the phosphate stretching band of DNA/RNA and the peak at 1573 cm^−1^ assigned to the ring breathing modes of the DNA/RNA bases, in the spectrum acquired from the equatorial segment covering about the 50% of the sperm nucleus (black line in Fig. 5b). To confirm that the spectral differences between AA and ES spectra are attributed to the biochemical differences discussed above, PCA was performed on the AA and ES spectra and results summarized in Fig. S2. A good separation can be achieved by using the first three PCs, suggesting that there is an inherent molecular difference between the two analyzed head regions. The ES region appears to have a higher level of nuclear material when compared to the AA region, as represented by PC1 of Fig. S2.

**Figure 7:**
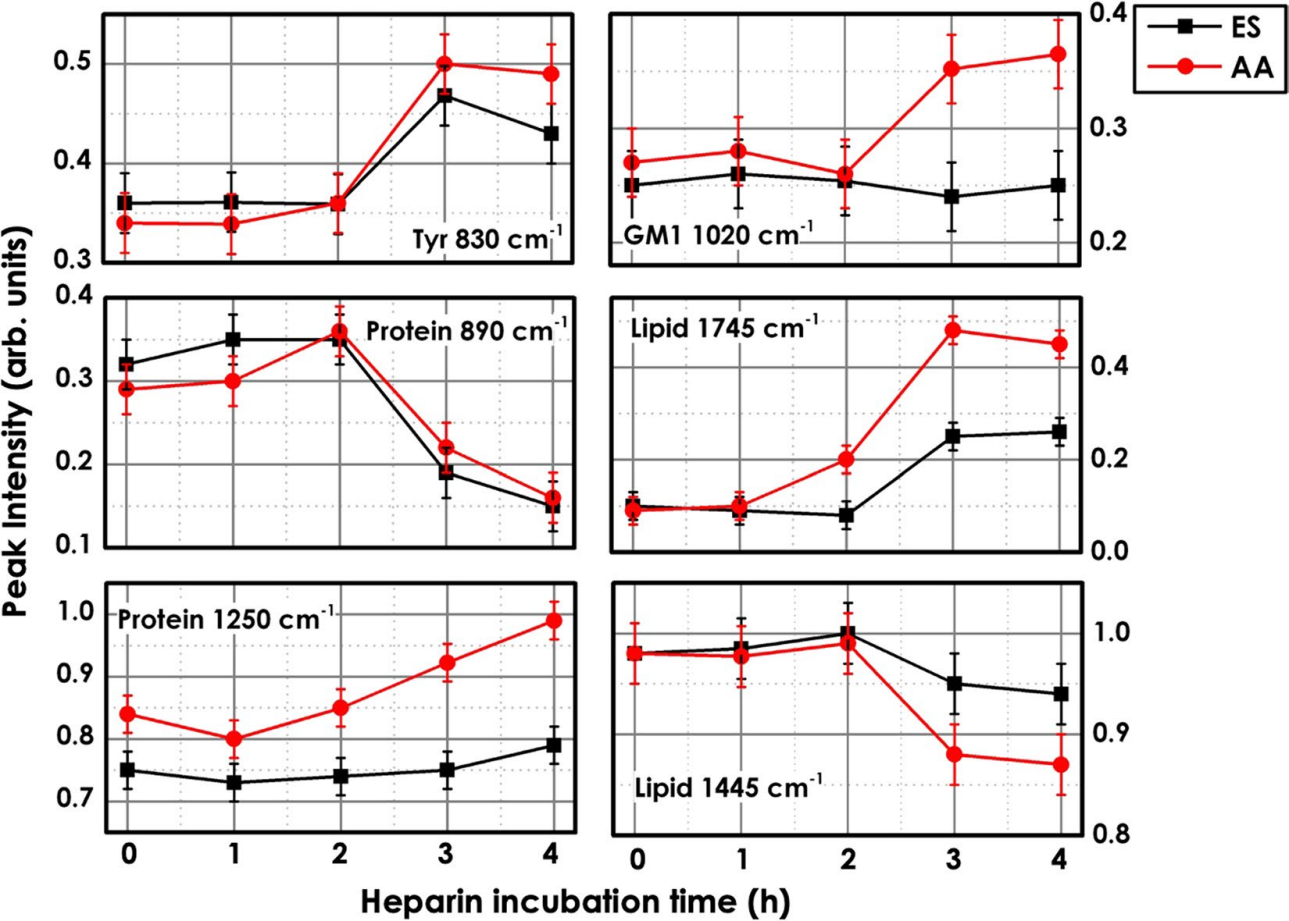
Effect of heparin on the intensity of the tyrosine residue peak at 830 cm^−1^, the protein bands at 890 and 1250 cm^−1^, the phospholipid peak (GM1) at 1020 cm^−1^ and the lipid bands at 1745 and 1445 cm^−1^. Black lines and markers correspond to data from the equatorial segment (ES) region and red lines and markers to data from the apical acrosomal vesicle (AA).

The heparin exposed samples have been analyzed by RM. In Fig. 6a, we compared the average spectra from AA and ES cell regions after the treatment with heparin for 0 (control sample), 1, 2, 3 and 4 hours of incubation time. After 1 and 2 hours of treatment, no significant spectral changes can be detected in both the cell regions compared to the control sample (0 h). As expected, the most important variations occurs after 3 and 4 hours, reflecting the cellular response to heparin. In the Raman spectra from AA region variations in the protein peak intensities are observed. Indeed, the bands at 830 (tyrosine residue), 1250 (*β*-sheet amide III), 1420–1445 (CH in proteins/lipids), 1480 (amide II), 1560 and 1573 cm^−1^ (tryptophan), 1609 and 1670 cm^−1^ (amide I) associated with protein content increase after 4 h of incubation time. In the ES region, as shown in Fig. 7, the intensity of the protein bands smoothly increase, and at the same time a spectral shift of the band associated with Amide III and Amide I can be detected. On the other hand, the isolated Raman band at 890 cm^−1^, previously assigned to glycoproteins^45^, strongly decreases in both cell regions (see Fig. 7). Indeed, the trigger event for capacitation seems to be the loss of decapacitation factors (glycoproteins) that coat the sperm membrane. Other important spectral changes can be detected in the lipid spectral region (1020; 1300–1375; 1420–1445 and 1745 cm^−1^). The bands exclusively due to the lipid content, showing a low overlapping with protein spectral region, such as the bands between 1420–1445 cm^−1^ decrees in capacitated sperm cells, especially in the AA region, probably indicate the dispersion of acrosomal vesicle during acrosome reaction. A different behaviour can be measured for the signal at 1745 cm^−1^, assigned to the stretching vibration of the C=O group (see Fig. 7). Interestingly, the peak associated to the glycosphingolipid^46^ (GM1) at 1020 cm^−1^ increased in the AA region (not in the ES region).

**Figure 8:**
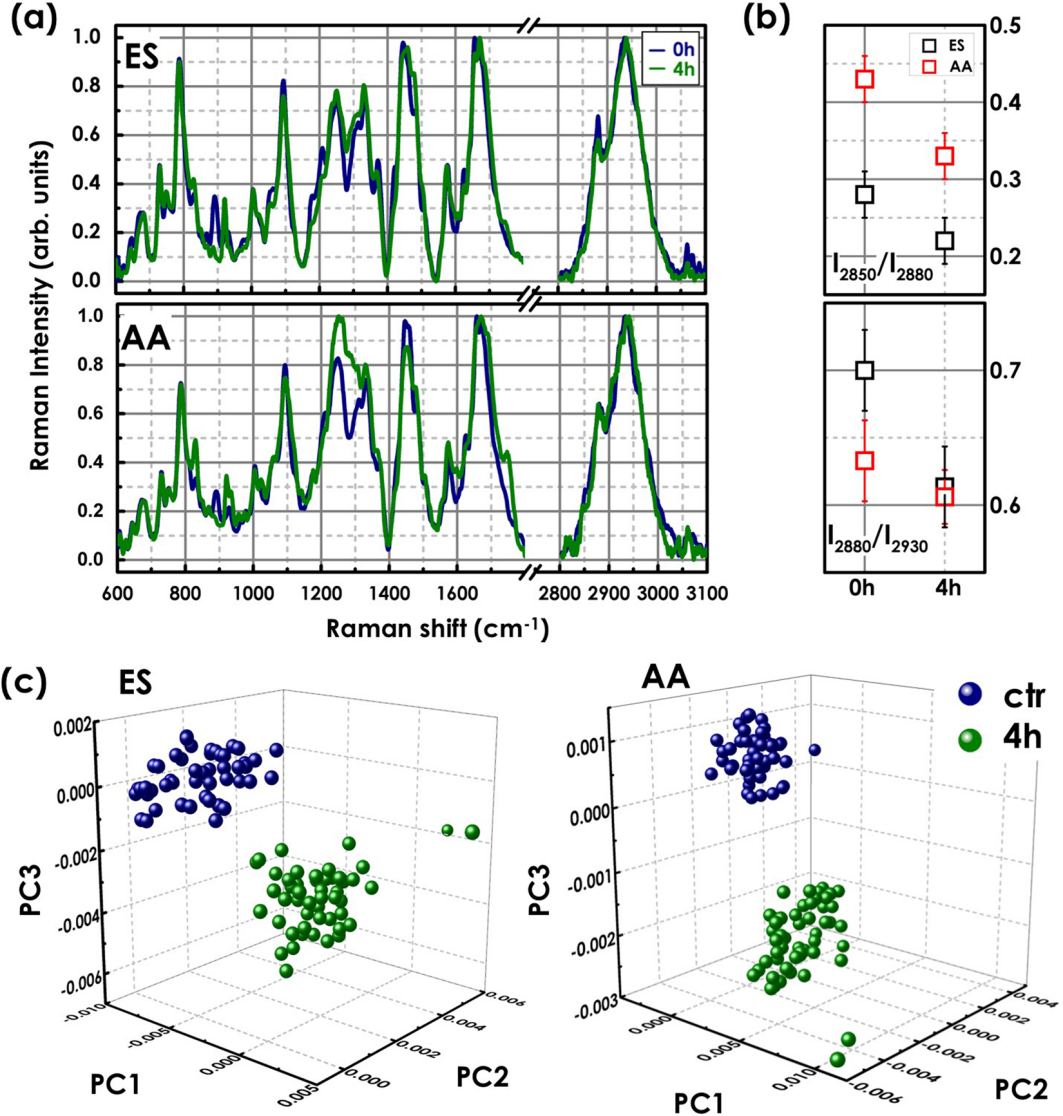
(**a**) Mean Raman spectra from the AA and ES sperm cell regions comparing control sample (0 h) and capacitated sperm cells (4 h in heparin). (**b**) *I*_2850_/*I*_2880_ and *I*_2880_/*I*_2930_ ratio variation in capacitated sperm compared to the control sample in the AA and ES regions. (**c**) 3D PCA scatter plots of the first three principal components for each the sperm cell region exposed to heparin for 0 (ctr) and 4 h. The Raman spectra corresponds to cells preliminarily selected by DHP analysis.

Figure 8a directly compares the Raman spectra from the regions AA and ES for 0 (control sample) and 4 hours of heparin treatment, to better identify the spectral differences between capacitated and non-capacitated sperm cells. The CH spectral region between 2800–3100 cm^−1^ is additionally shown. The molecular dynamics of the membrane lipids and proteins can be additionally characterized by monitoring the symmetric and asymmetric stretching modes of hydrocarbon chain methylene around 2850 cm^−1^ and 2880 cm^−1^. Indeed, the intensity ratio between these two bands, *I*_2850_/*I*_2880_, reflects the variation in inter-chain interactions (lateral packing order) within lipid assemblies^47,48^. Figure 8b shows that the ratio *I*_2850_/*I*_2880_ decreased from 0.43 and 0.28 for non-reacted sperm cells to 0.33 and 0.22 for reacted sperm cells, in AA and ES, respectively. The decrease in cholesterol/phospholipid ratio was in agreement with expected cholesterol efflux during capacitation, that causes phospholipid disorders and induces a higher fluidity to cell membrane. Interestingly, the intensity of 2880 cm^−1^ did not change in the AA spectrum, while strongly decreasing in the ES spectrum, suggesting a migration of lipids from the equatorial segment to the apical acrosome.

To identify Raman spectroscopic changes that correlate with different phases of the cell capacitation response, PCA was performed on the entire Raman spectra data set inclusive of all time points (0, 1, 2, 3, 4 hours) of heparin treatment. Figure 6c shows the scatter plots of the first three principal components (PC1, PC2, PC3) for both cell regions, describing 72%, 20% and 5% of the spectral variance between the AA spectra and 69%, 20% and 3% for the ES spectra. Raman spectra of cells exposed to heparin for 0, 1 and 2 hours are strongly localized in one cluster. Detectable spectral changes appears after 3 h (magenta dots) and 4 h (green dots), when a statistically significant number of sperm cells (about 80% by the CTC test) are capacitated and both the cell regions reveals similar results. Cross validation was performed using the leave-one-out approach and the results summarized in the confusion matrix of Fig. 6d. The corresponding loading of PC1, PC2, PC3 are shown in the supplementary information (Fig. S3). The PC1 loadings show negative lipid features at 1445 cm^−1^, indicating lower contributions in capacitated cells towards positive PC1, which are the control spectra (0 h). Negative features can be found in the glycoprotein region at 890 cm^−1^. Positive features in the protein spectral regions characterize the loadings of PC2 and PC3.

The PCA analysis have been repeated on the Raman spectra of the sperm cells un-capacitated (0 h) and capacitated (4 h in heparin) preliminary analyzed by birefringence. Figure 8c, shows the scatter plot of the first three principal components for both the cell regions for 0 and 4 hour of incubation with heparin. The first three principal components account for the 81%, 15% and 2% of the total variance for AA spectra and about 78%, 17% and 3% for ES spectra. The two clusters, corresponding to capacitated and un-capacitated cells, are now completely separated. The cross validation analysis revealed that PCA can correctly classified all the two cell types into their respective cluster. Two spectra, showing spectral variation in DNA region, are completely separated from the others, as they probably correspond to damaged cells.

## Discussion

Using a multi-modal system we have acquired simultaneously both a quantitative birefringence information and Raman signature that can assess the sperms ability to respond to capacitation stimuli (sperm function). Here, it has been used to evaluate the sperm capacitation induced *in vitro* by exposing sperm from healthy donors to heparin for different times (1, 2, 3 and 4 hours).

The acrosome reaction was evaluated by analyzing the birefringence of the sperm cell samples, using a HI setup. In a birefringent sample, the transmitted light undergoes a phase change on the two orthogonal field components, thus the birefringence property can be investigated through the phase difference in the orthogonal components of light crossing the specimen. Because of longitudinally oriented protein filaments, human sperm cells are naturally birefringent when observed under polarized light. A relation between the birefringence of the sperm head and its state of healthy was already demonstrated^17^, as well as between birefringence and the acrosome reaction^19,20^. The total birefringence of the sperm head reflects its health and DNA integrity, while a partial or absent birefringence is linked to pathological problems, in which the incidence of abnormalities in the inner protoplasmic structures is high^17^. On the other hand, the birefringence pattern changes from the intact acrosome state (non reacted - total birefringent head) to the acrosome reaction state (reacted - birefringence only in post-acrosomal region)^19,20^. Thus, the *in vitro* capacitation process of semen by heparin can be applied only in selected sperm cells with a completely birefringent head, ensuring that their DNA is not fragmented. Then, the reacted sperm cells can be selected by studying their birefringence pattern and choosing those that have birefringence only in the post-acrosomal region. This process can potentially increase in-clinical pregnancies in patients with severe male factor infertility^17,19,20^. HI has been demonstrated to be a valid tool for the polarization state of birefringent sample analysis^34–36^. Since HI can measure and localize the polarization state of an object, in this work it has been used to measure the birefringence in the control (0 h in heparin) and in treated (4 h in heparin) sperm samples. Results showed a high increase in the number of cells with a birefringence pattern confined to the post-acrosomal region with respect to the number of cells with a total birefringent head when passing from acrosome intact to reacted acrosome, confirming the potential of the proposed method for the selection of reacted sperm cells. Moreover, a better discrimination has been demonstrated using the information deriving from the phase difference that can be evaluated with the PSDHI microscope, with respect to the information obtained from the amplitude ratio that can be achieved also with a standard polarized microscope, confirming the potential of the proposed method for the selection of reacted sperm cells. However, there is no ideal solution for all problems, and the birefringence analysis for sperm function assessment also has its limitations. Indeed, the sperm cells with partial birefringent heads have been revealed and correlated with other cell characteristics^20^. HI when applied to sperm samples is only able to identify intact and reacted acrosomes without necessarily providing the molecular specificity. Thus to obtain molecular information (or indeed any information concerning the biochemical content), other techniques must be employed.

To provide additional information to that provided by birefringence/morphological measurements, we incorporated the HI into a multimodal system, where Raman and holographic measurements are made on the same sample. Integrating HI and RM allowed us to simultaneously characterize the morphology and chemical content of the sample without labels and with sub-cellular resolution. As such, RM is well suited to correlate biochemical variations with different phases of the capacitation response. RM allows discrimination between cells based on their molecular composition. Here, it has been used to evaluate the sperm cell capacitation induced *in vitro* by exposing sperm cells from healthy donors to heparin for different times (1, 2, 3 and 4 hours). The main spectral differences between the capacitated and uncapacitated cells arise from protein/lipid Raman profile reflecting the biochemical changes occurring during capacitation and the acrosome reaction.

The trigger event for capacitation is the loss of surface proteins (glycoproteins, known as decapacitation factors) coating the cell membrane. Indeed, the Raman analysis reveals that the glycoprotein peak around 890 cm^−1^^45^ decreases after 2 h of treatment with heparin. The capacitation is accompanied by a time-dependent increase in the protein tyrosine phosphorylation especially in the acrosomal region^5,7^ and a re-distribution of lipids in the plasma membrane. As a matter of fact, the Raman analysis highlighted several changes in the Raman bands of tyrosine residues associated with tyrosine phosphorylation^49^ in the AA region of the sperm cell. Indeed, the band at 830 cm^−1^ strongly increased in intensity. Additionally, the protein band at 1250 cm^−1^ increases in the AA region, and smoothly increases in the ES region. At the same time a spectral shift of the band associated with Amide III and Amide I can be detected. These features may be due to protein changes and replacement. Amide III, in particular, is very sensitive to changes in the main chain conformation in membrane proteins^41^. Indeed, the observed shifts in the amide III (1250 cm^−1^), amide II (1480 cm^−1^) and amide I (1670–1690 cm^−1^) bands suggests that the tyrosine phosphorylation may promote a *β*-sheet-like structure, as previously reported^49^.

The bands resulting from lipid content show a low overlapping with the protein spectral region, such as the bands between 1420–1445 cm^−1^ decreasing in capacitated sperm cells, especially in the AA region, probably indicating the dispersion of the acrosomal vesicle during the acrosome reaction. A different behavior can be seen for the signal at 1745 cm^−1^, assigned to the stretching vibration of the C=O group, probably due to lipid oxidation. Interestingly, the peak associated with the ganglioside GM1 (1020 cm^−1^)^46^, a glycosphingolipid used in the Cap-test as marker^15^ for assessing the capacitation status in sperm cells, increased in the AA region (not in the ES region). Both these features are probably due to the redistribution of lipids and phospholipids from the ES towards the entire acrosomal head following capacitation event^15^.

Moreover, the increase in the intensity of the peaks related to proteins (1250 cm^−1^) and oxidized lipids (1745 cm^−1^) revealed the formation of lipid rafts at the apical ridge of the sperm head containing sphingolipids, cholesterol, oxidized lipids and epididymal proteins such as GPI anchored proteins^8^. Out from these microdomains, cholesterol is oxidized (enhanced intensity of the band at 1745 cm^−1^) and removed from the sperm surface by albumin.

Raman analysis clearly detected this event in the spectrum at a higher wavenumber where in both the investigated sperm regions (ES and AA) the peak at 2850 cm^−1^ (characteristic of cholesterol) decreased. The intensity ratio between the peaks at 2850 and 2880 cm^−1^ is an empirical parameter serving to index the strength of lateral interchain packing interactions^47,48^. A reduction in its value reflects a decrease in the spacing between lipid chains^47^, as expected with lipid raft formation. Complementary information come from the intensity ratios of the complex Fermi resonance features at 2930 and 2880 cm^−1^, indicating changes in intrachain conformational order^47^. The increase of this latter parameter, in the ES sperm region, may indicate stronger lateral chain-chain interactions, with a higher degree of intrachain conformational order, suggesting a considerable interdigitation into the opposite lipid leaflets, typical of the membrane domains that are enriched in sphingolipids (i.e. lipid rafts)^50^. The overall lipid/phospholipid content (I_2930_/I_2880_ ratio) remained almost constant in the AA region, suggesting a migration of lipids from the equatorial segment to the apical acrosome. This was also confirmed by the increase of the glycolipid GM1 peak at 1020 cm^−1^ mainly in AA spectrum.

To correlate the spectral variations with the time-dependent cell capacitation response, the entire Raman spectra data set inclusive of all time points (0, 1, 2, 3, 4 hours) with heparin treatment was analyzed by PCA. For each cell subset, no significant spectral variation can be detected between different donors. Further, Raman spectra of cells exposed to heparin for 0, 1 and 2 hours are strongly localized in one cluster, for both AA and ES cell regions. Detectable spectral changes appear after 3 and 4 hours, when a statistically significant number of sperm cells (about 80% by the CTC test) are capacitated. Interestingly, the data set from the cells exposed to heparin for 2 h displayed a slight overlap (about 15% in AA region) with data from the cell exposed to heparin for 3–4 h as summarized in confusion matrix of Fig. 6d, confirming the results of the CTC test. This overlap is less than the 5% for the cell exposed to heparin for 1 h.

The PCA analyses were repeated on the Raman spectra of un-capacitated sperm (0 h) and capacitated sperm (4 h in heparin) preliminary analyzed by birefringence. In this case, the two clusters corresponding to capacitated and un-capacitated cells are completely separated. Two spectra are isolated from the others, as they probably correspond to damaged cells. Indeed, the spectral fingerprints of the two selected cells showed a lower signal-to-noise ratio and a variation in DNA-specific signature bands^39^. Clearly, the combined Raman and birefringence results suggest that the multimodal approach allows us to further divide the sperm cells into three subgroups: capacitated, un-capacitated and damaged.

In conclusion, the DH microscopy alone can provide quantitative information on the cell morphology and motility, as shown in our previous works^23,32^. The RS approach, on the other hand, it can provide complementary specific biochemical fingerprint of the sample, without affecting the integrity of living cells^51^. Here, we combined RS and PSDHI for a complete and accurate assessment of fixed air-dried semen in a label free manner. The novelty of the present work is the possibility to add characterization of the undergoing reaction of the acrosome by combining the study of the Raman spectra and the polarization state. Indeed, considering our findings, a new fully label free protocol for the selection of healthy and reacted sperm cells can be implemented. Choosing by PSDHI sperm cells with a head that is totally birefringent, their integrity is ensured;^20^ then, on these selected cells, the heparin treatment can be applied in order to trigger the acrosome reaction. Only the effectively reacted spermatozoa will be selected for ICSI by characterizing again their polarization state by PSDHI combined with the study of their Raman spectra. This combined characterization guarantees the selection of sperm cells in which the change in their birefringence pattern can be ascribed only to the acrosome reaction, removing those in which this change is associated with defects. Further studies on live sperm should be undertaken to validate the detected spectral bands and birefringence results and their use as markers of capacitation. Once optimized and stringently assessed, the proposed multimodal non-invasive model could provide the means of identifying and selecting capacitated and undamaged sperm for use in a clinical environment.

## Materials and Methods

### Sample preparation

Semen samples were donated, with written informed consent, from men attending the Centre of Assisted Fertilization (CFA, Naples). All ejaculates were provided by masturbation after 3–5 days of sexual abstinence and processed after liquefaction (37 °C, 30 minutes). The sperm used for experimentation was excess material not used for clinical purposes and was normally discarded. The experimentation on human spermatozoa were performed in accordance with relevant guidelines and regulations approved by the ethical committee of Centre for Assisted Fertilization, Naples, Italy. According to the criteria for normal concentration and morphology established by the World Health Organization (WHO), the semen was subjected to a Percoll density gradient to enable the preliminary separation of motile from non-motile spermatozoa and other cells present in the seminal fluid (see Supplementary Information).

The spermatozoon were divided into four aliquots: one untreated, as our control sample, to the others we added 100 g/ml of heparin at 37 °C for 1 hour, 2 hours and 4 hours, respectively^11,12^.

For the Raman and holography experiments, the sperm samples were washed twice in PBS for 5 min and incubated in a formaldehyde (3, 7%) fixing solution at 4 °C (30 min). Aliquots of 10 *μ*l of the sperm suspensions were smeared onto CaF_2_ coverslip (Crystran LTD, 150 *μ*m thickness), air-dried and sealed with a CaF_2_ slide, by using a common nail polish.

### Vitality and motility test

Before and after heparin exposure, sperm motility was assessed by using a Makler counting chamber (SAFI Medical Instruments). To assure that the treatment with heparin did not affect sperm cell viability, an eosin-nigrosin staining vitality test was performed before and after treatment (see Supplementary Information).

### CTC test

The chlortetracycline (CTC) test was performed as previously described by Fraser *et al*.^43,44^ with a minor modifications counterstained with DAPI (1 *μ*g/ml). The CTC stock solution was prepared by dissolving 750 *μ*mol/l di CTC-HC1 (Sigma) in a buffer containing 130 mmol/l di NaCl, 5 mmol/l cysteine, 20 mmol/l Tris (pH 7.8) and stored in a light-shielded container at 4 °C. A 100 *μ*l aliquot of sperm suspension was added to 100 *μ*l CTC solution and mixed gently. Cells were then fixed by 20 *μ*l of 37% formaldehyde. After incubation, 10 *μ*l of sperm suspension was placed on a glass slide, smeared, in Vectashield antifade mounting medium and overlaid by a cover slip. Samples were examined with a Nikon Eclipse E1000 fluorescent microscope (excitation at 490 nm and emission at 525 nm) equipped with a 100X objective; images were acquired with a CCD camera (Applied Imaging 4912–5010) and processed using Genikon Image software (NIKON). A total of 100 sperm cells per slide were examined within few hours. Sperm cells were evaluated according to CTC staining patterns^43,44^: fluorescence over the entire head (uncapacitated cells), fluorescence-free band in the postacrosomal region (capacitated cells) and low fluorescence over the entire head except for a thin bright fluorescent band along the equatorial segment (acrosome-reacted cells).

### Multimodal polarization sensitive digital holographic imaging and Raman microscope

The birefringence and spectral analysis of the sperm samples was carried out by the multimodal microscope shown in Fig. 2a. The polarization sensitive digital holographic microscope is designed for transmission imaging and consists in two Mach-Zender interferometers, one for each reference beam (*R*1 and *R*2). The laser source was a He-Ne emitting at *λ* = 633 nm (Thorlabs, Max power = 21 mW in CW, coherence length ≈60 cm). An objective lens (*OBJ*1, Zeiss, 10X, 0.22 N.A.) collects the laser beam and sends it into a single mode optical fiber; then the laser beam is split through an optical fiber coupler (YF, Single Mode Fiber Coupler, 1 × 2, 70/30, FC/APC, Newport) into object and reference beams. The collimated (*C*1, Thorlabs F240APC-B) object beam (*Obj*) is linearly polarized by a polarizer (*P*1 oriented at 45°) and a quarter waves plate (*QWP*1 oriented at 0°); then, the transmitted wave from the sample is collected by an objective lens (*OBJ*2, 60X, Olympus, water immersion, NA = 1.2). Thus, the *Obj* state of polarization can change when passes through the specimen, taking into account the sample birefringence proprieties. *R*1 and *R*2 beams are orthogonally linearly polarized by a polarized beam splitter (*PBS*), while quarter waves plates (*QWP*2 and *QWP*3) ensure the maintainance of linear polarization, avoiding interference between them. *Obj*, *R*1 and *R*2 beams are then overlapped by the beam splitter *BS*1 and the interference between the object and references waves yields the polarization digital hologram that is recorded by a CCD (CCD1, AVT Marlin F145B2, 1392 × 1040 pixel array; pixel dimensions Δx = Δy = 4.7 *μ*m) in an off-axis configuration, with the three waves propagation along different directions (see the inset in Fig. 2a). The half waves plates *HWP*1 and *HWP*2 allow to adjust the intensities of *Obj*, *R*1 and *R*2, while the mirrors *M*2 and *M*3 control the angles of incidence of *R*1 and *R*2, respectively. The excitation laser at 785 nm (Sacher Laser, TEC420, 1W), used as Raman probe, was reflected by the dichroic mirror (DM) and focused on the sample through an inverted microscope (Olympus IX51), equipped with an objective lens (60x, Olympus, water immersion, NA = 1.2). The laser power on the sample was 5 mW, to avoid cellular photodamage. The back-scattered light was collected and collimated by the same objective lens, and filtered by a holographic notch filter (HNF, Semrock, LPD01–785RU) in order to remove the Rayleigh scattering contribution. The Raman signal was focused onto the entrance slit (set at an aperture of 100 *μ*m) of the monochromator (Acton SP2300, Princeton Instruments) equipped with a 600 gr/mm holographic grating, and finally detected by a back-illuminated CCD camera (Pixis:400BR-eXcelon; Princeton Instruments), thermoelectrically cooled at −70 °C. The entrance slit aperture in combination with the objective defines a cylinder of examination in the focal plane with diameter ~0.7 *μ*m and depth of ~1.5 *μ*m. Spectral resolution of the system was around 2 cm^−1^.

### Hologram acquisitions and data analysis

At the CCD surface, the hologram intensity distribution is:

1 $$\begin{array}{rcl}H(x,y) & = & (Obj+R1+R2)\cdot {(Obj+R1+R2)}^{\ast }\\  & = & |Obj{|}^{2}+|R1{|}^{2}+|R2{|}^{2}+ObjR{1}^{\ast }+ObjR{2}^{\ast }+Ob{j}^{\ast }R1+Ob{j}^{\ast }R2\end{array}$$

In Eq. 1, the first three terms correspond to the zero diffraction order, the fourth and fifth terms produce the virtual images, and the last two terms form the real images. The polarization state of the object wave can be retrieved by the polarization hologram reconstruction; with this aim, the Fourier transform of the acquired hologram is performed and, by selecting two different spatial filter on the spectrum, the spatial frequencies of the real image are selected separately^34–36^. The two filtered and complex holograms, corresponding to the orthogonal components of the object field (*Obj*_*x*_ and *Obj*_*y*_), can be then obtained by performing the inverse Fourier transform of the spatial frequency components selected and applying the standard reconstruction algorithm^34–36^. By knowing the two orthogonal complex fields, it is possible to retrieve the amplitude map and the phase map for each component. With this aim, a proper Matlab code was implemented. Finally, in order to study the birefringence property of the sample, typically two parameters are considered: the amplitude ratio *β*, which gives information on the different transmitted intensities related to the two orthogonal components, and the phase difference Δ*ϕ*, that takes into account the different optical paths due to the anisotropy of the refractive index. Both the polarization parameters should be considered to obtain the distributions of the Jones vector at the surface of the specimen with a single image acquisition^34^. The phase difference and the amplitude ratio parameters can be expressed as:

2 $$\begin{array}{rcl}{\rm{\Delta }}\varphi  & = & phase(Ob{j}_{x})-phase(Ob{j}_{y})+{\rm{\Delta }}{\varphi }_{R}\\ \beta  & = & arctan(\frac{|Ob{j}_{x}|}{|Ob{j}_{x}|})\end{array}$$

where Δ*ϕ*_*R*_ = *phase* (*R*2) − *phase* (*R*1) can be compensated by a preliminary calibration^34^. Regarding the evaluation of the *β* parameter, we assume that the intensities of the reference waves are equal (|*R*_1_| = |*R*_2_|); this is obtained by adjusting the orientation of the half waves plates (HWP1 and HWP2 in Fig. 2a)^36^.

30 cells for the control sample (0 h) and the sample treated with heparin for 4 h were analysed. The experiment was repeated on semen collected from three different healthy donors. Data were first analysed by differentiatiating the three birefringence patterns (total, partial, abnormal) in both the amplitude ratio and phase difference maps. Then, in order to avoid errors due to the operator, a mask was applied to both the amplitude ratio and phase difference maps to highligthing only the head of each examined sperm cell. Two set of vectors were generated considering the histogram representing the number of pixel with a specific grey level. PCA was conducted on the two datasets obtained on the two considered maps.

### Raman acquisitions and data analysis

Two cell regions - the equatorial segment, ES, and the apical acrosome, AA (see the cell structure shown in Fig. 5a)- were analyzed for the Raman experiments. The control sample (0 h), and the samples incubated in heparin for 1, 2, 3 and 4 hours were analyzed. The experiment were repeated on the semen collected from three different healthy donors.

Each Raman spectrum was acquired with a 5 s integration time. A Matlab script was used to align the spectra to compensate for any small drift in the laser wavelength over the experimental period. The Raman spectra were background corrected by subtracting a baseline, estimated by a third-order polynomial fit. The data were normalised according to total area under the Raman spectrum.

PCA was used to analyse and classify the Raman data^38,52,53^. This multivariate statistical approach reduces the entire complex spectra to only a few significant components - the Principal Components, PCs - by finding combinations of the original dimensions that represent the largest variations between the data sets. The PCA was performed on the Raman data from both the control sperm cells and the sperm cells incubated with heparin for different times. The first three components were selected as they accounted for the major variance in the dataset (>95%). The score and loading plots were used to identify the main spectral variation spectral variations between samples and identify discrete data groups correlating with different phases of the cell capacitation response. The accuracy of our approach in correctly identifying the sperm capacitation from its spectrum, was determined using the leave-one-out cross validation procedure. One spectrum is left out from the data set and all the remained spectra used to construct a diagnostic algorithm. The procedure was repeated for all the acquired spectra, and the predicted values used to build up the confusion matrix^38,52,53^.

## Supplementary information

Supplementary Information

## Data Availability

The datasets generated during and/or analysed during the current study are available from the corresponding authors on reasonable request.

## References

[1] Khatur A, Rahman MS, Pang M-G (2018). Clinical assessment of the male fertility. Obstet Gynecol Sci.

[2] Kerns K, Zigo M, Drobnis EZ, Sutovsky M, Sutovsky P (2018). Zinc ion flux during mammalian sperm capacitation. Nat Commun.

[3] Bedford J (1983). Significance of the need for sperm capacitation before fertilization in euterian mammals. Biol Reprod.

[4] Leahy T, Gadella BM (2011). Sperm surface changes and physiological consequences induced by sperm handling and storage. Reproduction.

[5] Naz RK, Ahmad K, Kumar R (1991). Role of membrane phosphotyrosine proteins in human spermatozoal function. J Cell Sci.

[6] Visconti PE (1995). Capacitation of mouse spermatozoa i. correlation between the capaciation state and protein tyrosine phosphorylation. Development.

[7] Barbonetti A (2010). Protein tyrosine phosphorylation of the human sperm head during capacitation: immunolocalization and relationship with acquisition of sperm-fertilizing ability. Asian J Androl.

[8] van Gestel RA (2005). Capacitation-dependent concentration of lipid rafts in the apical ridge head area of porcine sperm cells. Mol Hum Reprod.

[9] Badawy S, Shue F, Chohan KR (2006). Sperm capacitation: effect of assisted reproductive technology. Middle East Fertil Soc J.

[10] Mansour RT (2008). The impact of spermatozoa preincubation time and spontaneous acrosome reaction in intracytoplasmic sperm injection: a controlled randomized study. Fertil Steril.

[11] Valecia A, Wens MA, Merchant H, Reyes R, Delgado NM (1984). Capacitation of human spermatozoa by heparin. Arch Androl.

[12] Parrish JJ, Susko-Parrish J, Winer MA, First NL (1988). Capacitation of bovine sperm by heparin. Biol Reprod.

[13] DasGupta S, Mills CL, Fraser LR (1993). Ca(2+)-related changes in the capacitation state of human spermatozoa assessed by a chlortetracycline fluorescence assay. J Reprod Fertil.

[14] Medoza C, Carreras A, Moos J, Tesarik J (1992). Distinction between true acrosome reaction and degenerative acrosome loss by a one-step staining method using pisum sativum agglutinin. J Reprod Fertil.

[15] Moody MA (2017). Validation of a laboratory-developed test of human sperm capacitation. Mol Reprod Dev.

[16] Collodel G (2010). Natural sperm birefringence can be used to estimate sperm viability and morphology. Syst Biol Reprod Med.

[17] Gianaroli L (2008). Sperm head’s birefringence: a new criterion for sperm selection. Fertil Steril.

[18] Garrido, N. & Rivera, R. A practical guide to sperm analysis-basic andrology in reproductive medicine. *CRC Press, 1*^*st*^*edition* (2017).

[19] Gianaroli L (2010). Birefringence characteristics in sperm heads allow for the selection of reacted spermatozoa for intracytoplasmic sperm injection. Fertil Steril.

[20] Magli MC (2012). Head birefringence properties are associated with acrosome reaction, sperm motility and morphology. Reprod Biomed Online.

[21] Di Caprio G (2010). Quantitative label-free animal sperm imaging by means of digital holographic microscopy. IEEE J Sel Top Quantum Electron.

[22] Crha I (2011). Digital holographic microscopy in human sperm imaging. J Assist Reprod Genet.

[23] Coppola G (2014). Digital holographic microscopy for the evaluation of human sperm structure. Zygote.

[24] Haifler M (2015). Interferometric phase microscopy for label-free morphological evaluation of sperm cells. Fertil Steril.

[25] McReynolds N, Cooke FGM, Chen M, Powis SJ, Dholakia K (2017). Multimodal discrimination of immune cells using a combination of Raman spectroscopy and digital holographic microscopy. Sci Rep.

[26] Kang JW (2011). Combined confocal Raman and quantitative phase microscopy system for biomedical diagnosis. Biomed Opt Express.

[27] Pavillon, N., Hobro, A. J., Akira, S., & Smith, N. I. Noninvasive detection of macrophage activation with single-cell resolution through machine learning. *Proc Natl Acad Sci USA*, 10.1073/pnas.1711872115 (2018). 10.1073/pnas.1711872115 PMC5866539 29511099

[28] Ferrara MA (2016). Simultaneous holographic microscopy and Raman spectroscopy monitoring of human spermatozoa photodegradation. IEEE J Sel Top Quantum Electron.

[29] Ferrara MA (2015). Label-free imaging and biochemical characterization of bovine sperm cells. Biosensors.

[30] De Angelis A (2017). Combined Raman spectroscopy and digital holographic microscopy for sperm cell quality analysis. J Spectrosc.

[31] Aknoun S, Aurrand-Lions M, Wattellier B, Monneret S (2018). Quantitative retardance imaging by means of quadri-wave lateral shearing interferometry for label-free fiber imaging in tissues. Opt Commun.

[32] Di Caprio G (2014). 4D tracking of clinical seminal samples for quantitative characterization of motility parameters. Biomed Opt Express.

[33] Daloglu MU (2018). Label-free 3D computational imaging of spermatozoon locomotion, head spin and flagellum beating over a large volume. Light Sci. Appl..

[34] Colomb T (2002). Polarization imaging by use of digital holography. Appl Opt.

[35] Colomb T, Cuche E, Montfort F, Marquet P, Depeursinge C (2004). Jones vector imaging by use of digital holography: Simulation and experimentation. Opt Commun.

[36] Palacios, F. *et al*. Phase and polarization contrast methods by use of digital holographic microscopy: Applications to different types of biological samples. *Holography - Basic Principles and Contemporary Applications* (2013).

[37] De Luca AC, Dholakia K, Mazilu M (2015). Modulated Raman spectroscopy for enhanced cancer diagnosis at the cellular level. Sensors.

[38] Manag‘o S (2016). A reliable Raman-spectroscopy-based approach for diagnosis, classification and follow-up of b-cell acute lymphoblastic leukemia. Sci. Rep..

[39] Mallidis C (2011). *In situ* visualization of damaged DNA in human sperm by raman microspectroscopy. Hum Reprod.

[40] De Luca AC (2014). Non-invasive sex assessment in bovine semen by Raman spectroscopy. Laser Phys Lett.

[41] Li N, Chen D, Xu Y, Liu S, Zhang H (2014). Confocal Raman micro-spectroscopy for rapid and label-free detection of maleic acid-induced variations in human sperm. Biomed Opt Express.

[42] Sanchez V (2012). Oxidative DNA damage in human sperm can be detected by Raman microspectroscopy. Fertil Steril.

[43] Fraser LR, Abeydeera LR, Niwa K (1995). Ca(2+)-regulating mechanisms that modulate bull sperm capacitation and acrosomal exocytosis as determined by chlortetracycline analysis. Mol Reprod Dev.

[44] Fraser LR (1998). Modulation of mammalian sperm function by fertilization promoting peptide (fpp). Androl.

[45] Ashton L, Pudney PDA, Blanch EW, Yakubov GE (2013). Understanding glycoprotein behaviours using raman and raman optical activity spectroscopies: Characterising the entanglement induced conformational changes in oligosaccharide chains of mucin. Adv Colloid Interface Sci.

[46] Basu I, Mukhopadhyay C (2015). In silico phase separation in the presence of GM1 in ternary and quaternary lipid bilayers. Phys Chem Chem Phys.

[47] Mendelsohn R, Moore DJ (1998). Vibrational spectroscopic studies of lipid domains in biomembranes and model systems. Chem Phys Lipids.

[48] Hu Z (2015). Raman spectroscopy for detecting supported planar lipid bilayers composed of gangliosidegm1/sphingomyelin/cholesterol in the presence of amyloid-b. Phys Chem Chem Phys.

[49] Xie Y, Zhang D, Jarori GK, Davisson VJ, Ben-Amotz D (2004). The Raman detection of peptide tyrosine phosphorylation. Anal Biochem.

[50] Stillwell, W. An introduction to biological membranes - (second edition). *Elsevier, 2nd Ed* (2016).

[51] Edengeiser E (2005). Non-invasive chemical assessment of living human spermatozoa. RSC Adv.

[52] Notingher I (2005). Multivariate analysis of Raman spectra for *in vitro* non-invasive studies of living cells. J Mol Struct.

[53] De Luca AC (2014). Reproducible surface-enhanced Raman quantification of biomarkers in multicomponent mixtures. ACS Nano.

