# Supplementary material for: Combined Raman and polarization sensitive holographic imaging for a multimodal label-free assessment of human sperm function
Source: Sci Rep. 2019 Mar 18;9:4823. doi: 10.1038/s41598-019-41400-0 (PMC6423271; doi:10.1038/s41598-019-41400-0)
Supplement: Supplementary file 1 — Supplementary Information [file 41598_2019_41400_MOESM1_ESM.pdf]

Supplementary Information

Combined Raman and polarization sensitive holographic imaging for a multimodal label-free assessment of human sperm function

Annalisa De Angelis[1], Maria Antonietta Ferrara[2], Gianfranco Coppola[3], Loredana Di Matteo[3], Laura Siani[3], Brian Dale[3], Giuseppe Coppola[2,*,‡], and Anna Chiara De Luca[1,*,‡]

[1]Institute of Protein Biochemistry, National Research Council of Italy, Via P Castellino 111, Naples, 80131, Italy
[2]Institute for Microelectronic and Microsystems, Unit of Naples, National Research Council of Italy, Via P Castellino 111, Naples, 80131, Italy
[3]Centro Fecondazione Assistita (CFA-Italia), Via Manzoni 15, Naples, 80123, Italy

[*]giuseppe.coppola@cnr.it;a.deluca@ibp.cnr.it
[‡]these authors contributed equally to this work

Results and discussion

Sperm vitality and mobility.
The viability test was performed on samples of seminal fluid from three normal donors with an age between 30 and 48 years, before, after density gradient centrifugation (Percoll) and after heparin (4h) treatment. A clear increase in the resistance of viable spermatozoa was considered following the Percoll gradient. In particular, spermatozoa showed a vitality, before treatments, of about 50% (± 6%). The Percoll gradient protocol, described in the Methods, allowed to select viable sperm (after Percoll gradient: 90% ± 5%, after heparin treatment: 90% ± 6%); after the treatment with heparin the spermatozoa presented about the same percentages of vitality.
Experiments conducted for evaluating the sperm motility after Percoll gradient and after heparin treatment indicated that motility is not affected by heparin treatment. In particular, before the Percoll gradient there is a percentage of progressive motile spermatozoa (medium/fast) of 52% ± 3%, non-progressive motile (slow) 19% ± 2 % and non-motile 29% ± 3%. After the heparin treatment, the motility remains about the same (progressive motile: 51% ± 3%, non-progressive motile 18% ± 2%; properties: 31% ± 2%).

Polarization sensitive digital holographic imaging of sperm capacitation. Being quantitative phase microscopy, polarized digital holography allows us to retrieve the average phase difference $\Delta\varphi$ obtained for control (0h in heparin) and reacted (4h in heparin) samples.

These values show a decrease of about fourfold of the mean value of Δφ when the acrosome reaction occurs. Results are summarized in the following table:

Supplementary Table 1. Mean value of phase difference evaluated on the ROI of all the examined cells.

	0h in heparin	4h in heparin
**Mean(Δφ) [rad]**	1.39	0.36

Regarding the PCA, this analysis was performed on the amplitude ratio and phase difference data sets obtained for control (0h in heparin) and reacted (4h in heparin) samples. Figure S3 shows the loadings of PC1, PC2, PC3 for both amplitude ratio (β) and phase difference (Δφ) parameters. PC1 indicates that a large contribution to discrimination is related on part of cell that is not birefringent; this can be seen by the large contribution from the histogram bin at 0 radiant, which represents the masked pixels and those have Δφ=0 and β=0. This is in good agreement with results of β and Δφ maps reported in Fig. 3-a and 3-b. PC2 and PC3 can distinguish more subtle differences between different phase of cells capacitation.

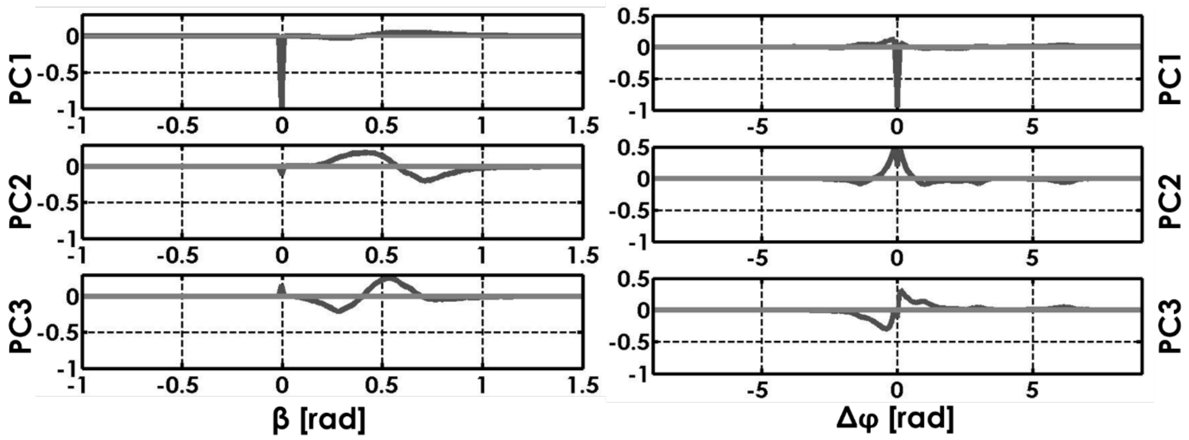

Supplementary Figure 1. PCA Loading of the first three principal components for both amplitude ratio (β) and phase difference (Δφ) parameters.

Raman Analysis of the sperm capacitation.

Raman spectra obtained from the AA and ES sperm head regions were preliminary analysed. To identify the spectral variation differences between the spectra acquired from these cell regions, we performed the PCA. The score plot and the corresponding PC1, PC2 and PC3 loadings for the spectra acquired from the ES and AA cell regions are reported in Figure S2. A good separation can be achieved by using the first three PCs, suggesting that there is an inherent molecular difference between the two analyzed head regions. The ES region appears to have a higher level of nuclear material when compared to the AA region, as represented by PC1. The accuracy of the algorithm, determined using the leave-one-out cross validation approach, was about 95%.

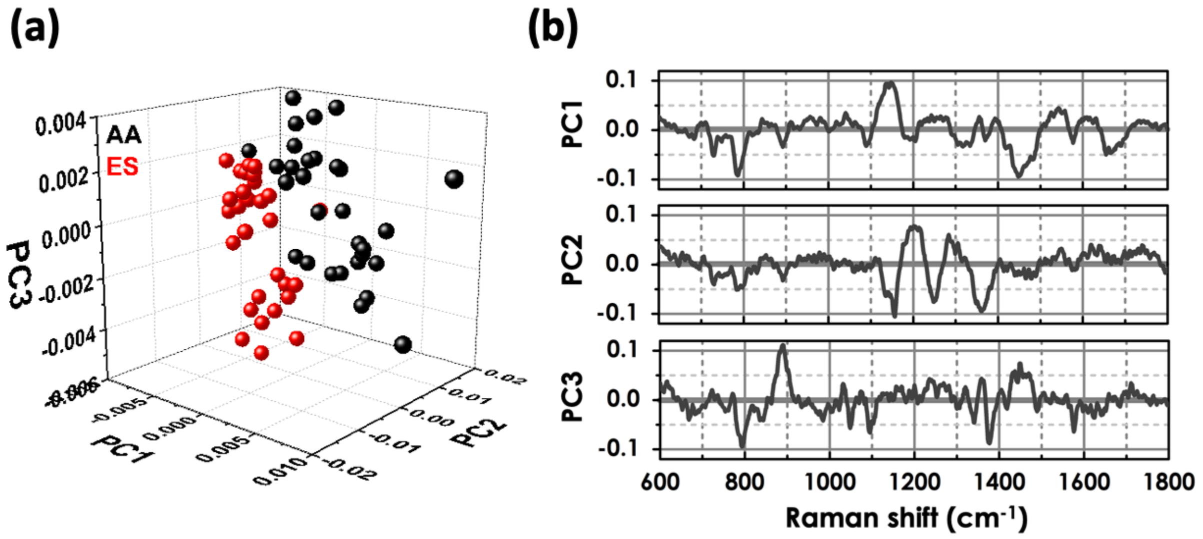

(c)

	Predicted class	
Actual class	AA	ES
AA	93%	7%
ES	3%	97%

Supplementary Figure 2. (a) PCA score plot and (b) loading of the first three principal components for the spectra acquired from AA and ES cell regions. (c) Confusion matrix.

To identify Raman spectroscopic changes that correlate with different phases of the cell capacitation response, PCA was performed on the entire Raman spectra data set inclusive of all time points (0, 1, 2, 3, 4 hours) of heparin treatment. Figure S3 show the loadings of PC1, PC2, PC3 for both ES and AA cell region. The PC1 loadings show negative lipids features at 1445 cm^{-1}, indicating a lower contribution in capacitated cells towards positive PC1, which are the control spectra (0h). Negative features can be found in the glycoprotein region at 890 cm^{-1}. Positive features in the protein spectral regions characterize the loadings of PC2 and PC3.

Methods

Sample preparation. The Percoll density gradient was prepared as follows: 1 ml of 80\% Percoll (SYDNEY IVF, K-SISG-50-80) was deposited in a sterile test tube with a conical bottom (15 ml). Subsequently, gently 1 ml of 55\% Percoll (SYDNEY IVF, K-SISG-50-40) was deposited taking care not to disturb the contact surface between the two layers. Sperm was then deposited on the top of the gradient and then centrifuged (300g, 20 min). The pellet obtained was resuspended in 2 ml of Ham's F-10 medium and centrifuged (300g, 5 min)

before resuspending, again, in 1-2 ml of Ham's F-10 medium, to obtain a sperm concentration of about 10 million sperm/ml.

Vitality and motility test. Before and after Percoll gradient and heparin treatment, sperm motility was assessed by using a Makler counting chamber (SAFI Medical Instruments). To assure that the treatment with heparin did not affect sperm cell viability, an eosin-nigrosin staining vitality test was performed before and after treatment. 20 µl of eosin (Carlo Erba - 446632) and 30 µl of nigrosin (Carlo Erba - 464853) were added to 10 µl of sperm sample for 30 s. Then, an aliquot of 10 µl of sperm was smeared and air-dried on a glass slide, and the sperm cells counted with a bright-field microscope equipped with a 100X objective. Spermatozoa with damaged membranes had a red or fark pink staining pattern over the entire head, while living spermatozoa (with intact membranes) showed a white staining pattern over the head. Vitality was evaluated as the percentage of spermatozoa with white fluorescence to the total of the observed sperm cells.

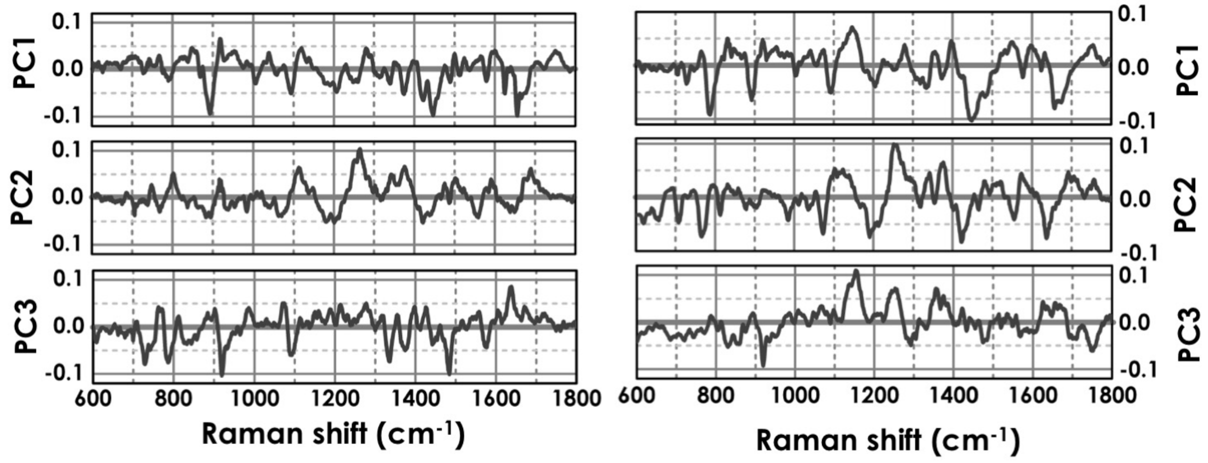

Supplementary Figure 3. PCA Loading of the first three principal components for both the cell regions.
